# Functional Gene Module–Based Identification of Phillyrin as an Anticardiac Fibrosis Agent

**DOI:** 10.3389/fphar.2020.01077

**Published:** 2020-07-17

**Authors:** Lei Wang, Wuxia Zhang, Ziwen Lu, Baofu Wang, Yang Li, Jingjing Yang, Peng Li, Mingjing Zhao

**Affiliations:** ^1^ Key Laboratory of Chinese Internal Medicine of Ministry of Education and Dongzhimen Hospital, Beijing University of Chinese Medicine, Beijing, China; ^2^ College of Arts and Sciences, ShanXi Agricultural University, Taigu, China

**Keywords:** cardiac fibrosis, gene expression profile, gene set enrichment analysis, marker genes, phillyrin

## Abstract

Cardiac fibrosis (CF) greatly influences the therapeutic effects of heart diseases and remains an urgent challenge in clinical therapy. Till now, only a few methods are used to find potential anti-CF drugs effectively. This study aimed to construct a gene functional module to represent the core pathological process of CF and screen antifibrotic agents capable of decreasing the expression of the gene functional module. First, three CF marker genes *Postn*, *Ddr2*, and *Pdgfra* were selected to identify the corresponding highest coexpressed genes in the genome-based transcriptional profiles of human hearts. Both the marker genes and the coexpressed genes formed the CF-related gene functional module. Second, the correlation of the module with the CF process was measured in a collection of gene expression profiles of heart diseases to evaluate the participation of the functional module in heart diseases. Third, the anti-CF effects of phillyrin were predicted by the enrichment analysis of the module in the phillyrin-induced transcriptional profile. Finally, the myocardial infarction animal model was used to validate the cardioprotective and anti-CF effects of phillyrin experimentally. The results showed that phillyrin was a novel antifibrotic agent in heart diseases.

## Introduction

Abnormal remodeling of heart tissue characterized by cardiac fibrosis (CF) is the core pathological change in the development of various cardiovascular diseases to a certain stage (Travers et al., 2016). Myocardial fibrosis involves the differentiation and proliferation of myofibroblasts and the secretion of large amounts of collagen, eventually resulting in a decline in myocardial compliance, ventricular arrhythmia, and even heart failure (Krenning et al., 2010). Therefore, inhibiting or reversing the CF process has become one of the important ways to treat cardiovascular diseases.

At present, two main drug development strategies are used for treating CF (Roubille et al., 2014; Gourdie et al., 2016). The first is from the perspective of etiology to target the primary disease or the cause of CF to improve the symptoms. For example, antihypertensive agents, including renin–angiotensin–aldosterone system (RAAS) inhibitors, β-adrenoceptor blockers, and calcium channel blockers, could alleviate the hypertension-induced left ventricular fibrosis in rat models. The drug effects were dependent on the relationship between the primary disease and fibrosis, as well as the severity of the disease. The second strategy was to target the CF process directly. For example, the development of antiinflammatory and immunoregulatory medications has a great potential for the CF treatment because the inflammatory response is an important initial factor of CF. In addition, molecular pathways, such as the transforming growth factor-β1 (TGF-β1) pathway associated with the activation and proliferation of cardiac fibroblasts, play a driving role in pathological ventricular remodeling and can be representative targets for the CF treatment.

However, most of the drugs targeting CF have encountered difficulties in clinical transformation, which may be attributed to the complexity of molecular mechanisms of fibrosis. Studies have revealed that CF involves a complex molecular network covering multiple biological molecules or pathways, such as the TGF-β1 pathway, RAAS pathways, inflammatory factors, endothelin, connective tissue growth factor, and platelet-derived growth factor (Leask, 2010). Drugs on individual targets hardly systematically delineate the fibrogenic network and may limit their therapeutic effects. Alternatively, a recapitulation of the molecular module of fibrosis could play a vital role in the development of anti-CF drugs. The present study aimed to construct a CF-related gene functional module (CFGM) based on the known CF markers and the genome-wide coexpression network. CFGM was built by integrating CF marker genes and the corresponding coexpression genes. Then, CFGM was used to evaluate the antifibrotic effects of drugs. Drugs that decrease the expression of most CFGM members have the potential to improve the fibrosis process.

**Figure 1:**
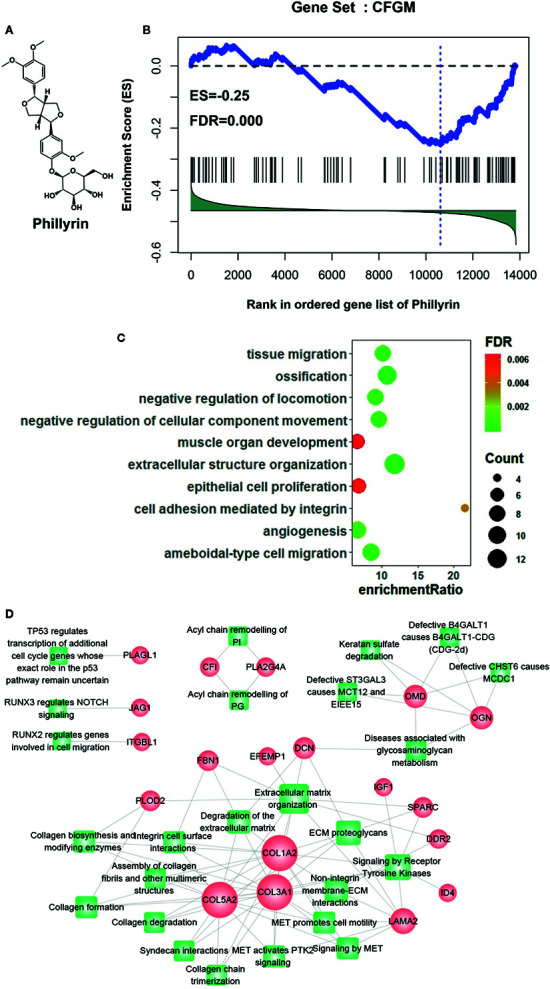
Gene set enrichment analysis of CF-related gene functional module (CFGM) member genes in the phillyrin-induced gene expression profile. **(A)** 2D structure of phillyrin. **(B)** CFGM was significantly enriched at the bottom of the phillyrin-induced gene expression profile. The plot of the running enrichment scores (*y*-axis) for CFGM in the phillyrin-induced gene list ranked with differential values in descending order (*x*-axis) has been shown. The black dashed line denotes zero of the running enrichment score. The black vertical lines indicate the location of CFGM members in the gene list. The blue vertical line shows the location of the maximum enrichment score. **(C)** Enrichment analysis of the Gene Ontology (GO) biological process for the core genes in CFGM regulated by phillyrin. **(D)** The gene-pathway network was generated from the 47 phillyrin regulated genes and their related pathways in Reactome database (FDR < 0.05). Nodes represent genes (shown as circles) and pathways (shown as rectangles). A link is placed between a gene and a pathway node if the gene is involved in the pathway. The size of the node is proportional to the number of the network degree.

*Forsythia suspense* (Thunb.) is a classical, traditional Chinese medicine (TCM) often used as an antipyretic and antiinflammatory drug. The investigations found that the extract of F. suspense (Thunb.) had many important pharmacological functions (Bai et al., 2015), and phillyrin (**Figure 1A**) is one of the major active constituents. Phillyrin has no acute or subacute toxicity in animal experiments (Han et al., 2017). It has exhibited important multitarget effects, such as antibacterial and antiinflammatory effects in the vitro and vivo studies (Diaz Lanza et al., 2001; Yang et al., 2017; Zhang et al., 2020). Phillyrin possessed appropriate pharmacokinetic properties with an oral bioavailability of 36.4% and drug-like 0.86 (TCMSP, http://tcmspw.com/tcmsp.php). Myocardial fibrosis is the most important pathological change after myocardial ischemia, and inflammation is one of the main factors inducing fibrosis. This study focused on the antifibrotic effect of phillyrin. Enalapril which has an antifibrotic effect was selected as the control medicine in this study (D’Souza et al., 2015; Ham et al., 2018). The antifibrotic effect of phillyrin was predicted based on its transcriptional profiles, which was further experimentally validated using animal models.

## Materials and Methods

### Collection of Transcriptional Data

The transcriptional data were collected from the National Center for Biotechnology Information (NCBI) Gene Expression Omnibus (GEO) and restricted to using only microarray data. The transcriptional data of human heart diseases were collected from three experiments (GEO accession: GSE3585, GSE57338, and GSE79962) with 376 samples, covering heart diseases of idiopathic dilated cardiomyopathy, ischemic heart failure, nonischemic dilated cardiomyopathy and septic cardiomyopathy. GSE3585 contains 12 left ventricular samples, collected from seven dilated cardiomyopathy patients at the time of transplantation and five nonfailing donor hearts which were not transplanted because of palpable coronary calcifications. GSE57338 contains 313 left ventricle samples, of them 177 are from patients with heart failure and 136 from nonfailing individuals. Among these heart failure patients, 82 are idiopathic dilated cardiomyopathy and 95 are ischemic heart disease. GSE79962 contains 51 left ventricular tissues, collected from 20 sepsis patients, 11 ischemic heart disease, 9 nonischemic dilated cardiomyopathy and 11 nonfailing donors. The transcriptional data of heart diseases of mice were obtained using (GEO accession: GSE775) the mouse model of myocardial infarction (MI), profiled in a time series (1 h, 4 h, 24 h, 48 h, 1 week, and 8 weeks).

The transcriptional data of phillyrin were obtained from a systematic study on TCM components (Lv et al., 2017). The human breast cancer epithelial cell line (MCF7) treated with phillyrin was measured for gene expression data using the microarray technology with Affymetrix Human Genome U133A 2.0 (GEO accession: GSE85871). For all transcriptional data, the raw data (CEL files) were processed consistently by applying the platform-specific procedure to filter and normalize data sets. Subsequently, for each condition, the differential gene expression profiles were calculated using the R package “Limma” (version 3.32.7) (Phipson et al., 2016). For each gene expression profile, the ordered gene list was built by ranking genes according to their differential expression values from small to large.

### Construction of CFGM

The CFGM was constructed by combining CF marker genes and the corresponding heart-specific coexpressed genes (**Supplementary Table 1**). Three genes *Postn*, *Ddr2*, and *Pdgfra* were selected as CF marker genes for their strong correlation with the activated cardiac fibroblast phenotype (Kaur et al., 2016). The coexpressed genes were identified in the Search-Based Exploration of Expression Compendium (SEEK, http://seek.princeton.edu/) using a query-level cross-validation–based algorithm (Zhu et al., 2015). In the heart tissue, the resulting genes a with coexpression *P* value <0.01 were included in the CFGM. Initially, both common *P* values 0.01 and 0.05 were examined. The CFGM contained 448 genes when *P <*0.05 compared with 135 genes when *P <*0.01, which directly affected the result of the subsequent enrichment analysis. Obviously, the stricter (smaller) the *P* value, the lower the corresponding enrichment score (absolute value). Compared with *P <*0.05, the selection of *P <*0.01 improved the criterion of predicting candidate drugs. Thus, a *P* value <0.01 was considered statistically significant.

### Enrichment Analysis of CFGM in Transcriptional Profiles

The characterization of CFGM in drug/disease-induced transcriptional profiles was evaluated using gene set enrichment analysis (GSEA), and implemented in the R package, “GSEA-P” (Subramanian et al., 2005). GSEA took CFGM and the order gene lists as inputs to calculate enrichment scores (ES) in gene expression profiles using the Kolmogorov–Smirnov statistics. The ES values within [−1 1] could evaluate the overrepresentation of CFGM at the extremes (bottom or top) of each transcriptional profile. The significance of ES values was estimated by comparing these with a set of ES_NULL_ values calculated randomly (Li et al., 2019).

### Leading-Edge Subset

A leading-edge subset means the core part of a gene set that accounts for the enrichment signal in GSEA analysis. The leading-edge subset of CFGM for each gene expression profile was extracted using the method implemented in the R packages, “GSEA-P”.

### Animals

All animal experimental protocols were performed in accordance with the Animal Care and Use of Laboratory Animals of the Beijing University of Chinese Medicine and met the requirements of laboratory animal management and use regulations. Male 8-week Sprague–Dawley rats, weighing 240 ± 10 g, were purchased from Beijing Vital River Laboratory Animal Technology Co. Ltd. [certification number SCXK (Beijing) 2016-0006]. The animals were fed a standard diet and water under the SPF-level conditions and subjected to a 12-h light/dark cycle, a temperature of 24°C ± 1°C, and a humidity of 60% ± 10%.

### Model Establishment and Grouping

After 5 days of acclimation, a model of myocardial fibrosis after MI was established as described in a previous study (Chen et al., 2019). The rats were anesthetized using intraperitoneal injections of 1% sodium pentobarbital (40 mg/kg). The animals were connected to an animal ventilator by endotracheal intubation, and the thorax was opened in the third intercostal space on the left. The left anterior descending coronary artery was ligated at 3 mm below the margin of the left auricle, between the pulmonary cone and the left auricle. The sham rats underwent the same procedure without ligation of the left coronary artery. In the successfully ligated rats, the electrocardiograph (ECG) showed six to eight pathological Q waves (including lead I, AVL, and V1–V6) on the second day after the surgery (Wang et al., 2017). Based on the pathological Q waves at this point in time, the ligated rats were randomly divided into four groups: the model group, the sham group, the phillyrin-treated group (phillyrin purchased from Chengdu Pufei De Biotech Co., Ltd; HPLC ≥98%; dose 100 mg/kg) (Zhou et al., 2016), and the enalapril-treated group (Yisu, H32026567; dose 2.86 mg/kg daily). The sham group was used as a negative control. Eight rats were used for each group. The rats were administered the corresponding drug *via* oral gavage from day 2 to day 30 after the surgery in the phillyrin and enalapril groups, while the rats in the sham and model groups were given drinking water at the same volume of 5 ml/kg.

### Measurement of the Heart/Weight Index

The body weights of the rats were measured before sacrificing them. Then, the hearts were collected quickly and photographed using a camera. The heart weight was tested and recorded after the removal of excess connective tissue. The heart/weight index (mg/g) was defined as the ratio of heart weight to body weight.

### ECG and Echocardiographic Examination

The cardiac structure and function were detected using the Vevo 2100 Imaging System Ultrasonic Diagnostic Equipment (Visual Sonics, Canada) with a 21-MHz high-frequency linear array transducer. The following indexes were detected: left ventricular anterior end-systolic wall (LVAWs), left ventricular anterior end-diastolic wall (LVAWd), left ventricular posterior end-systolic wall (LVPWs), left ventricular posterior end-diastolic wall (LVPWd), left ventricular internal end-systolic diameter (LVIDs), left ventricular internal end-diastolic diameter (LVIDd), left ventricular end-systolic volume (LVESV), left ventricular end-diastolic volume (LVEDV), ejection fraction (EF), and fractional shortening (FS).

### Hematoxylin–Eosin Staining

The heart was fixed in 4% paraformaldehyde for 48 h and cut horizontally at the maximum diameter. Then, a paraffin section was prepared routinely. The section was deparaffinized using xylene and then rehydrated using graded ethanol. A Hematoxylin–Eosin Staining Kit (G1120, Beijing, China) was used according to the manufacturer’s protocol as follows: staining with hematoxylin for 5 min, washing with deionized water, differentiation fluid differentiating for 10 s, staining with eosin for 1 min, and soaking in tap water for 3 min. Then, the slice was dehydrated with gradient alcohol for 2 min, rendered transparent with xylene for 20 min, and then sealed with neutral gum in turn. Finally, the stained tissue sections were observed under a microscope.

### Masson’s Trichrome Staining

The collagens in paraffin sections were stained using a Modified Masson’s Trichrome Stain Kit (G1345, Beijing, China) according to the manufacturer’s protocol as follows: incubation in Bouin solution at 37°C for 2 h, staining with celestite blue for 3 min, washing with deionized water for 10 s, staining with ponceau solution for 10 min, staining with a phosphomolybdic acid solution for about 6 min, staining with aniline blue for 20 min, dehydration with 95% and 100% ethanol for 2 min, making cells transparent using xylene for 20 min, and finally sealing the tissue slices with neutral resin. The collagen expression was quantified in 14 microscopic fields (eight survival area and six marginal area fields, *n* = 6 per group) chosen randomly at ×400 magnification. The collagen content was analyzed using Image-Pro Plus software. The collagen volume fraction (CVF) was equal to the ratio of the collagen area to the myocardial area.

### Statistical Analysis

All values were performed using the SPSS program package (SPSS version 20.0) and presented as mean ± standard deviation. One-way analysis of variance (ANOVA) and LSD and Tamhane’s T2 were used for comparisons. The significance of the overlapping analysis of the core genes between mouse and human samples was determined using the hypergeometric test. A *P* value <0.05 was considered statistically significant unless the context clearly dictated otherwise.

## Results

### Overexpression of CFGM in Transcriptional Profiles of Heart Diseases

A CFGM was constructed by combining CF marker genes and their coexpressed genes (**Supplementary Table 1**). Three genes *Postn*, *Ddr2*, and *Pdgfra* were selected as CF marker genes for their strongest correlation with the activated cardiac fibroblast phenotype (Kaur et al., 2016). The three marker genes were queried to SEEK to extract the corresponding highest coexpressed genes with a *P* value <0.01. CFGM contained a total of 135 heart-specific genes and was anticipated to be mostly relevant to the activation of the CF process in heart diseases.

CF is a core pathological change in the development of various cardiovascular diseases, including MI, hypertension, rheumatic heart disease, hypertrophic cardiomyopathy, and heart failure. Thus, CFGM genes should be significantly overexpressed in diseases involving CF. The gene expression data from different human heart disease samples were collected and analyzed using the GSEA method to validate the reliability of CFGM. From the GEO database, transcriptional data on human heart tissues of different heart diseases, including ischemic heart disease, nonischemic dilated cardiomyopathy, septic cardiomyopathy, idiopathic dilated cardiomyopathy, and ischemic heart failure, were collected. The analysis showed that CFGM members were significantly overexpressed in these diseases with high ES values (ES = 0.23–0.60, FDR = 0.00; **Table 1**). In addition, considering that phillyrin needed to be experimentally validated for its antifibrotic effects in animal models, the enrichment of CFGM was also tested in mouse samples. CFGM was characterized in transcriptional data of heart tissues of nice with MI produced by ligating the left coronary artery, which were profiled in a time series (1 h, 4 h, 24 h, 48 h, 1 week, and 8 weeks). The results showed that the CFGM genes were not significantly expressed at two early time points (1 h and 24 h, FDR >> 0.05; **Table 1**). This was reasonable because cardiac fibroblasts were activated in the infarcted region in the initial stage of CF; the expression of most genes related to CF was still insufficient. As expected, CFGM was significantly enriched in the gene expression profiles from 48 h to 8 weeks (ES = 0.18, 0.51, and 0.61; FDR = 0.04, 0.00, and 0.00 at 48 h, 1 week, and 8 weeks, respectively; **Table 1**) when the CF process was completely activated.

**Table 1:** Enrichment analysis of CF-related gene functional module (CFGM) in the gene expression profiles of heart tissues subjected to human heart diseases and mouse myocardial infarction model.

organism	GEO platform	GEO accession	Phenotype	ES	FDR
Homo sapiens	GPL96	GSE3585	Dilated cardiomyopathy	0.50	0.00
Homo sapiens	GPL11532	GSE57338	Idiopathic dilated Cardiomyopathy	0.50	0.00
			Ischemic heart failure	0.53	0.00
Homo sapiens	GPL6244	GSE79962	Nonischemic dilated cardiomyopathy	0.50	0.00
			Septic cardiomyopathy	0.23	0.00
			Ischemic heart disease	0.60	0.00
Mus musculus	GPL81	GSE775	1h myocardial infarction	0.13	0.20
			4h myocardial infarction	0.35	0.00
			24h myocardial infarction	0.14	0.14
			48h myocardial infarction	0.18	0.04
			1w myocardial infarction	0.51	0.00
			8w myocardial infarction	0.61	0.00

### Prediction of Phillyrin as a Candidate for Treating CF

CFGM can be used to find candidate drugs for targeting CF. The assumption is that drugs decreasing the expression of most CFGM members should suppress the CF process. In this study, the anti-CF effects of phillyrin were evaluated by examining the enrichment of CFGM in the phillyrin-induced transcriptional profile. Phillyrin was predicted to attenuate the CF process if CFGM genes most highly anticorrelated with phillyrin-induced transcriptional profiles. First, CFGM was constructed by combining three CF marker genes *Postn*, *Ddr2*, and *Pdgfra* and the corresponding highest coexpressed genes in the heart tissue (see Materials and Methods). Second, the phillyrin-induced gene expression profile was curated from the GEO database, and the ordered gene list was produced according to their differential expression values for further enrichment analysis. Finally, the enrichment of CFGM in the ordered gene list of phillyrin was measured using GSEA to assess its antifibrotic effects. As expected, the GSEA analysis indicated that phillyrin significantly decreased the expression of most CFGM members (**Figure 1B**; ES = −0.25, FDR = 0.00) and might be a candidate for treating CF.

Further, the functional association of the significantly influenced genes with CF was assessed in CFGM using phillyrin. The significantly regulated genes were the members in the leading-edge subset of the enrichment analysis (see Materials and methods). As shown in **Supplementary Table 1**, phillyrin could influence 47 genes in CFGM, including collagen genes (*COL1A2*, *COL3A1*, *COL5A2*, *COL15A1*, and *COL6A3*), fibrillin (*FBN1*), serpin family proteins (*SERPINF1* and *SERPINE2*), laminin (*LAMA2* and*LAMB1*), and lysyl oxidase (*LOX*). All these genes participated in the CF process. The phillyrin-regulated genes were characterized by the Gene Ontology (GO) enrichment analysis. The GO analysis showed that these genes were significantly involved in biological processes related to CF, such as “cell adhesion mediated by integrin,” “extracellular structure organization,” and “angiogenesis” (**Figure 1C**). The 47 phillyrin-regulated genes were analyzed by the pathway enrichment analysis in Reactome database (https://reactome.org). The result showed that these genes were significantly involved in 25 biological pathways related to 19 genes (these genes were found to be down-regulated). A gene-pathway network was constructed as shown in **Figure 1D**. In this network, 12 genes and 15 pathways compose the largest connected component of the network, reflecting a high degree of participation of phillyrin in related functions. From this network component, most of these proteins are related to scar formation. The network suggests that phillyrin mainly regulate functions related extracellular matrix formation and degradation. These analyses suggested that phillyrin could strongly influence the genes functioning in the fibrotic process in CFGM.

### Phillyrin Improved Heart Function and Structure

**Figure 2:**
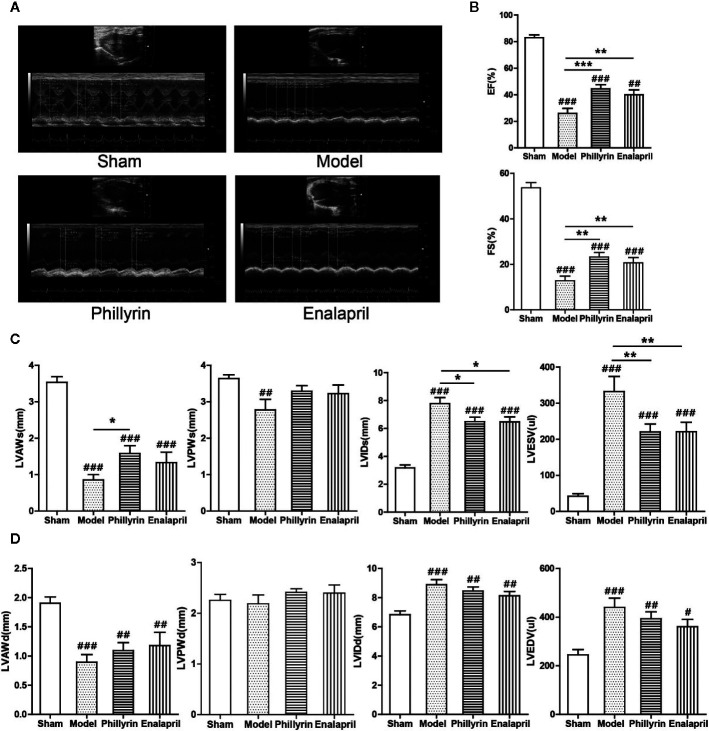
Effect of phillyrin on the cardiac function detected by echocardiography. **(A)** Echocardiography images of the sham group, model group, phillyrin group, and enalapril group. **(B)** ejection fraction (EF) and fractional shortening (FS) of the sham group, model group, phillyrin group, and enalapril group. **(C)** Myocardial systolic function assessments (LVAWs, LVIDs, LVPWs, and LVESV) of each group 30 days after the surgery. Myocardial diastolic function assessments (LVAWd, LVIDd, LVPWd, and LVEDV) in each group 30 days after the surgery **(D)**. ^#^ *P* < 0.05 or ^##^ *P* < 0.01 or ^###^ *P* < 0.001 versus the sham group; ^*^P < 0.05 or ^**^P < 0.01 or ^***^P < 0.001 versus the model group.

Phillyrin was predicted to be an antifibrotic agent that could improve cardiac function after MI. The EF, FS, LVAWs, LVAWd, and LVPWs values significantly decreased (**Figures 2A–D**) while LVIDs, LVIDd, LVESV, and LVEDV values significantly increased in the model group compared with the sham group (**Figures 2A, C, D**). No difference was found in LVPWd (**Figures 2A, D**). These data indicated the pathological changes in the model group in terms of the structure and function of the left ventricle. After 4-week treatment with phillyrin and enalapril, EF and FS obviously improved. Meanwhile, LVIDs and LVESV were lower but LVAWs was higher than those in the model group (There was no change in LVAWs in enalapril group). No appreciable changes were detected in LVAWd, LVPWd, LVIDd, LVEDV, and LVPWs (**Figures 2C, D**). These data suggested that phillyrin could improve heart function by regulating systolic indicators of myocardium.

### Phillyrin Attenuated the Fibrosis After MI

**Figure 3:**
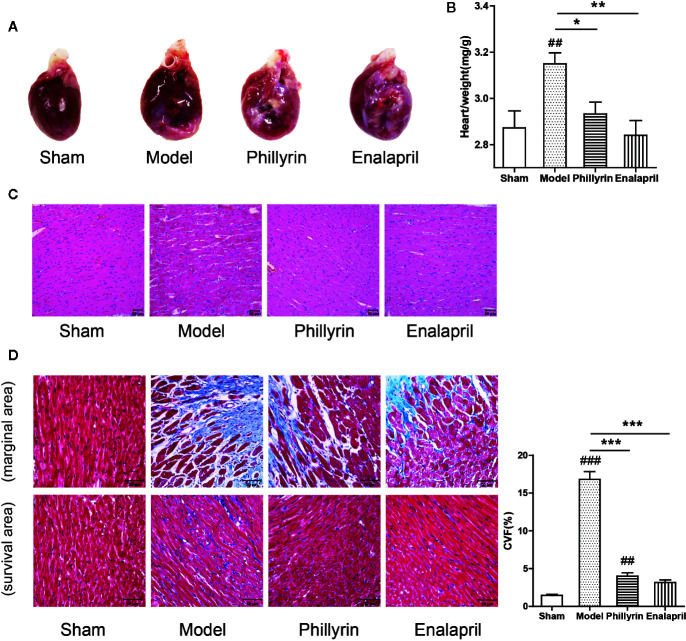
Effect of phillyrin on attenuating fibrosis detected by pathology and morphology. **(A)** Preparation of the hearts from the sham group, model group, phillyrin group, and enalapril group. **(B)** The heart/weight index was calculated by dividing the heart weight with the body weight. **(C)** Hematoxylin–eosin staining in each group 30 days after the surgery (magnification ×200). Scale bars, 50 μM. **(D)** Collagen was evaluated using Masson’s trichrome staining images and the collagen volume fraction (CVF) (magnification ×400). Scale bars, 50 μM. ^##^ *P* < 0.01 or ^###^ *P* < 0.001 versus the sham group; ^*^ *P* < 0.05 or ^**^ *P* < 0.01 or ^***^ *P* < 0.001 versus the model group.

Further, the antifibrotic ability of phillyrin was tested in MI animals. In the model group, the anterior ventricular wall was very thin and the necrotic myocardium was replaced by connective tissue to form a ventricular aneurysm. After 4-week of phillyrin treatment, the infarcted area and the aneurysm became smaller (**Figure 3A**). The heart/weight ratio significantly increased while phillyrin could reduce the ratio in the model group compared with the sham group (**Figure 3B**). Hematoxylin–eosin staining displayed that the myocardial cells were enlarged and disordered in the model group. After treatment with phillyrin, the cardiomyocytes in the infarcted area were more orderly and smaller (**Figure 3C**). Moreover, Masson’s trichrome staining was used to detect left ventricular fibrosis. The amount of collagen in the model group was higher than that in the sham group. Phillyrin could significantly reduce the content of the collagen (**Figure 3D**).

## Discussion

In the present study, a tissue-specific transcriptional module CFGM was annotated and integrated with fibrosis biomarkers and the corresponding coexpressed genes to delineate the biological module/network of CF. CFGM in the heart disease induced transcriptional profiles was analyzed, confirming the positive association of the fibrosis module with some heart diseases, including cardiomyopathy, heart failure, and MI. This study focused on phillyrin and revealed that phillyrin significantly decreased the expression of CFGM, suggesting that phillyrin might be a potential anti-CF agent. This was further validated using an animal experiment in which phillyrin attenuated the fibrosis process and improved the cardiac function and structure of rats with MI.

### Construction of the CFGM

CFGM in the heart disease-induced transcriptional profiles was analyzed, confirming the positive association of the fibrosis module with some heart diseases, including cardiomyopathy, heart failure, and MI (**Table 1**). In addition, the correlation between mouse and human data was examined by comparing the core genes of CFGM, which were significantly regulated in each sample. For each sample, the core genes were the members in the leading-edge subset of the enrichment analysis. The MI samples of 8 weeks were selected to represent samples in mice. **Supplementary Table 1** showed that, 41, 55, 63, 74, 58, 41, and 76 core genes were found in 8-week-old mice with MI, dilated cardiomyopathy (GSE3585), idiopathic dilated cardiomyopathy (GSE57338), ischemic heart failure (GSE57338), nonischemic dilated cardiomyopathy (GSE79962), septic cardiomyopathy (GSE79962), and ischemic heart disease (GSE79962), respectively. The overlapping analysis indicated that except idiopathic dilated cardiomyopathy (GSE57338) (hypergeometric test *P* = 0.10), core genes for MI significantly overlapped with those for other human heart diseases (**Supplementary Table 2**, hypergeometric test *P* < 0.05), implying that the MI in mice might share mechanisms of CF with other human heart diseases to some extent. This analysis had some limitations, indicating that the results should be interpreted with caution. First, this comparison was between different organisms (mouse and human), where many genes could not be mapped. Second, all these gene expression data were collected under different conditions, including sequencing platform, time, and sample source, thus influencing the results of data analysis greatly.

### Prediction of Anti-CF Effects of Phillyrin

CFGM could be used to exploit the anti-CF effects of drugs in the drug-induced gene expression profiles. This study focused on phillyrin, one of the main chemical ingredients of *F. suspense* (Thunb.), which was shown to be an important TCM. Phillyrin exhibits antiinflammatory (Lee et al., 2011), antioxidant (Wei et al., 2014), and antiobesity effects (Do et al., 2013). However, the impact of phillyrin against CF has not been reported to date. In the present study, an enrichment analysis method was used to predict the antifibrotic effects of phillyrin based on the phillyrin-induced gene expression profile in the MCF7 cell line.

MCF7 is a reference cell line in laboratories throughout the world, which has been extensively molecularly characterized. Despite the use of the MCF7 cell line, which is very different from the heart tissue, the analysis result was reasonable. Previous studies confirmed similar patterns of interdependency between transcription initiation and mRNA processing events of MCF7 in the brain, heart, and liver tissues (Anvar et al., 2018). Moreover, it was widely used in the study of obesity, Alzheimer’s disease, and immunity (Blanchard et al., 2004; Lamb et al., 2006; Subramanian et al., 2017). The gene expression profile is a common vocabulary used to translate diseases and drug action into the same language (Lamb, 2007). Studies have used the transcriptional profile to build functional connections between drugs and diseases (Iskar et al., 2013; Kidd et al., 2016; Subramanian et al., 2017). Moreover, MCF7 could greatly reduce the experimental overhead compared with the animal model. This was especially suitable for high-throughput screening and pilot drug testing. Finally, the phillyrin-induced transcriptional profile in the MCF7 cell line was only used to predict antifibrotic effects from the gene expression level. The actual efficacy of phillyrin still needs experimental validation.

### Phillyrin Improved the Heart Function and Attenuated the Fibrosis

The MI rat model induced by ligating the anterior descending branch of the left coronary artery is a classical model, which can replicate the pathological manifestations of CF. In this study, the results of echocardiographic examination, hematoxylin–eosin staining, and Masson’s trichrome staining showed that the MI model rats successfully replicated CF. In terms of heart function, phillyrin could significantly improve the value of EF, FS, and LVAWs and reduce the value of LVIDs and LVESV, which was consistent with the effect of enalapril. The results suggested that phillyrin was effective in regulating systolic indicators of the myocardium and ultimately improving cardiac function. In terms of the heart structure, smaller infarction area and aneurysm, lower heart/weight ratio, orderly and smaller cardiomyocytes, and less amount of collagen were found in phillyrin-treated rats. Previous studies confirmed the antifibrotic effects of enalapril. In this study, phillyrin showed effects similar to those of enalapril, further indicating that phillyrin might be a new type of anti-CF agent. Further, in this study, phillyrin was predicted to regulate many genes, including collagen genes, fibrillin, serpin family proteins, laminin, and lysyl oxidase, which are closely related to both fibrosis and inflammation (Van der Donckt et al., 2015; Jeong et al., 2018; Pastel et al., 2018; Choi et al., 2019; Leal-Lopes et al., 2019).

### Summary and Outlook

The results of this study provided evidence for the potential of the specific functional gene modules in drug development and suggested phillyrin to be a novel antifibrotic agent in heart diseases. However, the present results could not elucidate the concrete mechanism underlying the anti-CF effect of phillyrin. In future research, we aim at investigating the related molecular mechanisms mediating the anti-CF effect of phillyrin. We will5 select the phillyrin-regulated genes for qRT-PCR differential expression verification. Then the gene knockout or gene overexpression of the differential genes most relevant to CF will be applied. Animal experiments and molecular biology techniques will be conducted for further verification.

## Data Availability Statement

All datasets presented in this study are included in the article/**Supplementary Material**.

## Ethics Statement

The animal study was reviewed and approved by Animal Care and Use Committee of Beijing University of Chinese Medicine.

## Author Contributions

LW and WZ contributed equally to this study as co-first authors. The experiments were performed by LW, WZ, ZL, BW, YL, JY, MZ, and PL. PL and MZ conceived, designed, and funded the study. LW and WZ analyzed data and wrote and revised the manuscript.

## Funding

This work was supported by National Natural Science Fund (No. 81973787, No. 7161008, No. 81703945).

## Conflict of Interest

The authors declare that the research was conducted in the absence of any commercial or financial relationships that could be construed as a potential conflict of interest.
